# Beyond Principles: A Reflective-Cognitive Framework for Ethical Decision-Making in Anorexia Nervosa

**DOI:** 10.3390/healthcare14081047

**Published:** 2026-04-15

**Authors:** Evdoxia Tsigkaropoulou, Fragiskos Gonidakis, Ioannis Michopoulos

**Affiliations:** 1 Eating Disorders Unit, 1st Department of Psychiatry, Eginition Hospital, Medical School, National and Kapodistrian University of Athens, 11528 Athens, Greece; frgonid@med.uoa.gr; 2 Hellenic Society of Cognitive Psychotherapies, 11528 Athens, Greece; imihopou@med.uoa.gr; 3 Eating Disorders Unit, 2nd Department of Psychiatry, “Attikon” Hospital, Medical School, National and Kapodistrian University of Athens, 12461 Athens, Greece

**Keywords:** ethical decision making, anorexia nervosa, reflective thinking, cognitive theory, supervision

## Abstract

Anorexia nervosa is a clinically complex and ethically challenging psychiatric disorder. Clinicians are frequently confronted with ethical dilemmas arising from conflicts between core ethical principles in everyday clinical practice. Professional codes of ethics and legal frameworks often fail to provide a stable basis for resolving these dilemmas due to the fluctuating medical risk and the ego-syntonic nature of anorexia nervosa. Under conditions of heightened responsibility and medical risk, clinicians’ cognitive and emotional responses may be activated and may mediate ethical decision-making. Although such internal processes have been described in the literature, limited attention has been paid to their role in shaping ethical judgment in routine clinical care. The aim of this article is to conceptualize the decision-making processes that unfold in response to ethical dilemmas in the clinical context of anorexia nervosa and propose a sequential multi-level framework. A focused conceptual literature review was conducted to develop a reflective framework for clinical practice, drawing on selected studies in clinical ethics, healthcare law, anorexia nervosa care, and cognitive theory. Clinicians’ internal cognitive and emotional processes play a significant role in ethical decision-making in complex clinical contexts such as anorexia nervosa and should be explicitly recognized and brought into reflective awareness through supervision and reflective practice. Ethical decision-making is therefore conceptualized as a dynamic process linking clinical events, clinicians’ internal responses, ethical and legal considerations, and reflective clinical judgment. Incorporating structured reflection into clinical, educational, and supervisory settings may support more ethically informed and context-sensitive clinical judgment within multidisciplinary eating disorder services.

## 1. Introduction

Anorexia nervosa (AN) is among the most complex and ethically challenging psychiatric disorders, as it combines severe psychopathology requiring specialized psychotherapeutic intervention with potentially life-threatening medical complications that often necessitate intensive medical care [1,2,3]. The disorder is frequently associated with a chronic course, high relapse rates and elevated mortality [4,5]. Within this context, clinicians are repeatedly required to make decisions under conditions of marked uncertainty and heightened responsibility, while balancing the competing demands of medical safety and respect for patient autonomy [6,7].

A defining feature of AN is the need for integrated and often prolonged care, involving close collaboration between therapists, health professionals, and, frequently, family members. Contemporary treatment is therefore embedded within a broader framework of interdisciplinary practice, continuous risk assessment, and patient involvement in decision-making [8]. Emphasis on individualized treatment, informed consent, and the consideration of patients’ values and motivation constitutes a central principle of modern care, even in the presence of significant medical risk [9]. Psychotherapy remains a core component of treatment and is delivered by specialized clinicians applying theoretically and empirically informed approaches within clinical practice [3]. Evidence supports the effectiveness of psychodynamic [10], systemic family-based [11], and cognitive behavioral interventions [12]. In practice, however, treatment rarely involves a single clinician or a linear decision-making process. Rather, ethical and clinical decisions emerge at the intersection of multiple professional roles and responsibilities perspectives, rendering everyday clinical judgment in AN particularly complex [13].

Within this multifactorial clinical context, clinicians are repeatedly confronted with ethical conflicts such as autonomy versus protection of life and health, confidentiality versus safety, and the preservation of the therapeutic alliance versus the use of coercive or compulsory interventions. These conflicts are further intensified by features that are characteristic of AN, including the ego-syntonic nature of symptoms, marked ambivalence toward recovery, resistance to the involvement of others, and the fluctuating course of medical risk. As a result, ethical decision-making rarely involves isolated dilemmas that can be definitively resolved. Instead, clinicians navigate recurrent and evolving ethical challenges throughout the course of treatment, often in situations where uncertainty is unavoidable, and risk cannot be fully eliminated. Although professional ethical codes and legal standards—such as respect for autonomy, beneficence, nonmaleficence, and legal principles concerning decision-making capacity and consent—provide important normative guidance, they frequently prove insufficient for addressing the complexity and urgency of everyday clinical decisions in AN [7,14,15].

At the same time, ethical decision-making does not occur in a purely neutral space. Clinicians bring their own patterns of thinking, emotional responses, and moral values to each ethical dilemma. These internal processes are shaped by automatic thoughts, underlying schemas, cognitive biases, and emotional reactions, as well as by clinicians’ moral perspectives, prior knowledge and clinical experience, and are activated in situations involving risk, responsibility, and uncertainty [16,17]. Such processes may interact with ethical norms, legal considerations, and patients’ attitudes, influencing how ethical principles are interpreted and weighed in real time [18,19]. Consequently, ethical issues in AN do not arise as discrete events governed solely by external rules, but as ongoing challenges embedded within the therapeutic relationship and mediated by clinicians’ internal cognitive and emotional experience.

Existing literature has extensively examined ethical issues in AN from medical, legal, and bioethical perspectives, with particular emphasis on decision-making capacity, involuntary treatment, and risk management [7,14]. While prior work has acknowledged the cognitive processes and the emotional burden and moral distress experienced by healthcare professionals, comparatively limited attention has been paid to the role of clinicians’ internal cognitive processes in shaping ethical judgment during routine clinical practice [16,17,20]. Despite their substantial influence on clinical judgment, these processes often remain implicit and insufficiently examined.

Cognitive behavioral psychotherapy (CBT) for AN is grounded in a broader cognitive model of eating disorders and aims to modify dysfunctional thoughts and behaviors related to food, weight, and body image. Enhanced CBT (CBT-E) has demonstrated effectiveness in reducing eating disorder psychopathology and improving body mass index in patients with AN [12,21]. Cognitive-behavioral theory offers a well-articulated and operationalized framework through which clinicians may conceptualize and interpret ethical dilemmas. Importantly, the cognitive perspective adopted in this article is intended to provide a shared conceptual language for examining clinicians’ internal decision-making processes even across different therapeutic approaches.

To our knowledge, ethical decision-making in AN has not yet been systematically examined through a cognitive and reflective pathway that explicitly integrates clinicians’ cognitive and emotional responses. The present article aims to address this gap by offering a conceptual and narrative synthesis of the literature, on ethical decision-making in AN, focusing on adult patients and giving particular attention to these internal processes that may mediate the transition from ethical principles to clinical judgment.

## 2. Methods

The present article employed a conceptual review with elements of narrative synthesis to examine ethical decision-making in the clinical management of AN. A conceptual approach was selected because the aim of the study was not to systematically evaluate empirical evidence, but to integrate and interpret diverse bodies of literature—including clinical ethics, healthcare law, research on anorexia nervosa, and cognitive theory—in order to construct a theoretically informed framework. A targeted literature search was conducted using electronic databases including PubMed, PsycINFO, Scopus, Web of Science, and Google Scholar. The search strategy was guided by broad conceptual search terms, including “anorexia nervosa”, “ethical decision-making”, “clinical ethics”, “ethical dilemmas”, “codes of ethics”, “healthcare law”, “reflective thinking”, “cognitive theory”, “cognitive psychotherapy”, “therapist cognition”, “decision-making capacity”, “coercive treatment”, and “moral distress”. Keywords were chosen to reflect both clinical and theoretical dimensions of the topic and were iteratively refined during the literature review process to ensure relevance and coverage of the central concepts addressed in the article. Where applicable, Medical Subject Headings (MeSH) terms were used to enhance the sensitivity and specificity of the search. Combinations of keywords were applied in a flexible manner rather than through predefined search equations, consistent with the exploratory and conceptual nature of the review. In addition, a snowballing technique was employed by reviewing the reference lists of relevant articles to identify further key sources. This approach was intended to balance breadth and relevance, ensuring coverage of key conceptual domains while maintaining a focused and theoretically informed selection of sources.

The selection of literature was guided by conceptual relevance and applicability to clinical practice. Inclusion considerations included peer-reviewed publications in English, as well as influential and seminal works addressing ethical, legal, clinical, or cognitive aspects of decision-making in AN or related contexts. Exclusion considerations included studies not directly relevant to ethical analysis or clinical decision-making. In this sense, literature selection followed a purposive strategy aimed at identifying sources relevant to the broader ethical and legal context of AN, as well as those informing the analysis of clinicians’ cognitive and emotional processes in decision-making.

The analytical process followed an iterative and reflective synthesis. Initial identification of recurring ethical dilemmas in the literature informed the exploration of explanatory frameworks, particularly from cognitive theory. Through repeated engagement with the literature, key concepts such as automatic thoughts, schemas, cognitive biases, and emotional responses were integrated into a conceptual model. The framework was further refined through ongoing discussion among the authors, aiming to produce a coherent and clinically meaningful synthesis.

Specifically, first, ethical and legal standards were examined in terms of how they structure or constrain clinical decisions in routine practice. Second, recurring ethical dilemmas and decision-making challenges in anorexia nervosa were identified and thematically organized. Third, the limitations of principle-based approaches, such as those grounded in core ethical principles, in guiding complex clinical decisions were examined. Finally, insights from cognitive theory were applied to explore how clinicians’ internal cognitive and emotional processes, together with moral considerations, may influence ethical judgment when navigating such dilemmas. These levels of analysis were subsequently integrated into a reflective synthesis that conceptualizes ethical decision-making as a dynamic process in the context of AN.

## 3. Codes of Ethics and Legal Frameworks in the Care of Anorexia Nervosa

### 3.1. Codes of Ethics

Codes of ethics in healthcare practice provide a foundational context for clinicians, describing their responsibilities, obligations, and professional standards that guide relationships between health providers and their patients [22,23]. Across mental health disciplines, these codes emphasize core ethical principles, established over time, including respect for patient dignity, autonomy, beneficence, nonmaleficence, confidentiality and professional competence [24,25]. While ethical codes offer general normative guidance, their application requires interpretation within specific clinical contexts.

Given that the treatment of AN is largely delivered within psychotherapeutic frameworks, ethical principles in psychotherapy provide a directly relevant context for understanding clinical decision-making in this population. Within psychotherapy, ethical principles are applied by emphasizing respect for patients’ values and preferences, informed consent, protection of confidentiality, and the maintenance of clear professional boundaries [26,27,28]. Ethical guidelines also stress the therapist’s responsibility to recognize and manage situations involving increased risk, to seek consultation or supervision when appropriate, and to collaborate with other professionals involved when required for patient care. Ethical considerations have been central to psychotherapy since the early development of the field [29,30,31], while contemporary frameworks increasingly address challenges arising from societal changes and technological developments [32,33,34,35]. Ethics in psychotherapy are often closely connected to broader philosophical perspectives concerning human rights, autonomy, free will, and personal choice [36,37].

Although core ethical principles are widely shared across psychotherapy, different therapeutic approaches interpret and apply them within distinct theoretical frameworks. For example, psychodynamic approaches emphasize relational and implicit processes within the therapeutic relationship, while even within CBT, unconscious dynamics may be present, though not explicitly theorized. In the present article, a cognitive perspective is adopted as a shared conceptual framework for examining clinicians’ internal decision-making processes. Within CBT, ethical principles are integrated through an emphasis on the collaborative therapeutic relationship, commonly described as collaborative empiricism [38,39,40]. Cognitive approaches incorporate core ethical principles while highlighting patients’ active participation in treatment, respect for autonomy through informed choice, and transparency regarding therapeutic goals, roles, and responsibilities [39,41]. Patient ambivalence toward cognitive and behavioral techniques is addressed through informed consent to the therapeutic process, while ethical practice further requires careful attention to therapeutic boundaries and therapist self-awareness [42]. In parallel, broader social and cultural developments have influenced therapeutic practice, leading some clinicians toward a more authentic and participatory therapeutic stance [43]. Across contemporary psychotherapy, patient involvement in decision-making throughout the course of treatment has been increasingly emphasized, with informed consent and respect for autonomy identified as important contributors to positive therapeutic outcomes [34,44].

Although no ethical codes have been developed exclusively for AN, professional guidelines addressing eating disorders highlight the clinician’s responsibility to balance psychological treatment and respect for patient autonomy with attention to medical safety and protection from serious health risks [14]. These guidelines emphasize the importance of interdisciplinary collaboration, ongoing medical monitoring, and clear communication among professionals involved in care [45]. Ethical standards in the treatment of AN further highlight the need for informed consent, careful assessment of decision-making capacity, and appropriate documentation when elevated clinical risk is present [46,47].

### 3.2. Legal Principles and Standards

Although professional codes of ethics provide a stable normative foundation for clinical practice, legal principles also play a central role in shaping ethical decision-making in healthcare. Legal standards have often developed in response to adverse events or disputed clinical decisions, in which courts were required to assess whether patients’ rights were adequately protected and whether clinicians’ duties and obligations were appropriately fulfilled. Consequently, legal systems have evolved to establish standards of practice in high-risk contexts and to clarify the circumstances under which clinicians are authorized or required to intervene [48].

Legal considerations in mental healthcare were introduced to address the specific needs and complexities arising in the treatment of individuals with mental disorders particularly where decision-making capacity, risk, and treatment refusal are central concerns [49,50]. Although core bioethical principles are shared across healthcare systems, their legal interpretation and application may vary across jurisdictions, with some civil law systems placing greater emphasis on protective or paternalistic considerations, compared to the stronger focus on individual autonomy in common law traditions [23,51]. Within this context, forensic psychiatry emerged as a specialized field focusing on the interface between mental health practice and the legal system [52,53]. Treatment of AN has received particular attention within this field, given the frequent involvement of legal questions related to decision-making capacity, consent, and compulsory measures. Although specific legal provisions vary across countries, a set of common legal principles applies broadly to clinical decision-making in eating disorders and provides a relevant reference framework across healthcare settings [7,13].

Drawing on international literature from clinical practice, healthcare law, and empirical research, legal guidance in the field of eating disorders is commonly organized around several key domains, such as decision-making capacity and informed consent, refusal of treatment and compulsory intervention, confidentiality and information sharing, and clinicians’ duty of care.

### 3.3. Decision-Making Capacity and Consent

Decision-making capacity may be compromised in situations in which severe mental disorders or conditions affecting the central nervous system impair cognitive functioning, thereby limiting an individual’s ability to make judgments in their own best interests. A distinction should be made between decision-making capacity and competence: capacity is a clinical evaluation performed by healthcare professionals, while competence is a legal determination made by a court [52,54]. In mental healthcare, capacity is considered both task-specific and time-specific and may therefore fluctuate over time. In addition, individuals with mental disorders may exhibit cognitive rigidity and ambivalence, associated with their psychopathology, which are factors that can further interfere with decision-making capacity [54,55]. Decision-making capacity is closely linked to informed consent. Valid consent presupposes capacity, as individuals must possess sufficient decisional abilities to provide informed agreement to treatment. Informed consent requires that patients receive adequate information regarding the nature, risks, benefits, and alternatives of treatment, and that consent is given voluntarily and without coercion. Informed consent also requires that patients are supported in understanding that they have the opportunity to seek a second opinion, particularly in AN, where distrust and ambivalence toward treatment may be present [52,56]. In AN, the assessment of decision-making capacity is of particular importance, as patients may refuse treatment despite significant medical risk. Ongoing clinical assessment and reassessment are therefore required to identify factors that may compromise capacity, including cognitive impairment, or severe medical complications affecting cognitive functions. Legal standards emphasize careful and continuous evaluation of capacity and consent as essential components of lawful and responsible clinical practice [14,47]. In addition, the assessment of decision-making capacity is further complicated by ongoing debates regarding the status of illness-related beliefs, including whether extreme denial of illness severity or body image distortions may approximate delusional thinking. These considerations may influence both clinical and legal interpretations of capacity and treatment refusal [57].

### 3.4. Treatment Refusal and Compulsory Treatment

Legal systems generally recognize the right of competent adults to refuse medical treatment, even when such refusal may result in serious harm. However, this right may be restricted under specific conditions, particularly when a patient lacks decision-making capacity or when there is an immediate danger to life [56,58]. Compulsory treatment provisions have been developed to permit clinical intervention in situations where a severe mental disorder and significant risk are present. These laws aim to balance respect for individual autonomy with the protection of life and health within a structured legal framework. Compulsory treatment includes a spectrum of interventions, from involuntary hospitalization to forms of coercive outpatient care, often regulated under different legal frameworks [49,52]. In the context of AN, refusal of treatment is a common clinical challenge that may arise at any stage of the therapeutic procedure. The potentially life-threatening nature of the disorder, combined with concerns regarding patients’ decision-making capacity, gives rise to significant legal considerations [59]. In such cases, compulsory treatment or hospitalization may be legally sanctioned as a life-saving intervention [14,15,60].

Specifically, in AN, treatment refusal presents disorder-specific ethical and legal challenges that differ from other non-psychotic conditions, such as major depressive disorder, as refusal is often closely linked to ego-syntonic illness beliefs, diminished insight, and underestimation of medical risk. These features complicate the assessment of decision-making capacity and the interpretation of treatment refusal [61]. Consequently, compulsory treatment remains ethically controversial, particularly when high medical risk coexists with uncertain capacity [62]. Legal approaches vary across jurisdictions. For example, in Scotland, the concept of significantly impaired decision-making ability (SIDMA) allows intervention even when capacity is not entirely absent [63], whereas other systems apply different thresholds. In Spain, compulsory treatment has traditionally been more closely associated with severe behavioral disturbance, with anorexia nervosa representing a more ambiguous case [64]. These differences highlight the need for context-sensitive ethical and legal evaluation in AN.

### 3.5. Confidentiality and Information Sharing

Confidentiality is a fundamental principle in healthcare, protecting patients’ private information and fostering trust in the therapeutic relationship. Legal standards establish clear obligations for clinicians to safeguard confidentiality, while also defining specific circumstances under which disclosure may be permitted or required [28,56]. In mental healthcare, exceptions to confidentiality may apply when there is a serious risk of harm to the patient or others, or when disclosure is necessary for the provision of appropriate care [52]. In AN, decisions regarding information sharing frequently arise in the context of interdisciplinary collaboration, involvement of family members, and management of medical risk [13,45].

### 3.6. Duty of Care and Professional Accountability

Duty of care refers to the legal obligation of clinicians to act in accordance with accepted standards of practice to protect patients from potential harm [48,52]. In mental healthcare, this duty includes appropriate assessment, monitoring of risk, timely intervention, and collaboration with other professionals when necessary [65]. Professional accountability arises when clinicians are required to justify their clinical decisions and actions, particularly in high-risk situations [49,53]. In AN, duty of care extends beyond a simple symptom management to include attention to medical risk, coordination with interdisciplinary teams, and adherence to legal and professional standards [13,45]. In this context, duty of care is further complicated by the high risk of mortality associated with AN, including an elevated risk of suicide, which constitutes a significant proportion of deaths related to the disorder [5].

Taken together, ethical and legal requirements establish a structured normative context that defines acceptable professional conduct in the treatment of AN. They articulate core principles and responsibilities that guide clinical practice across therapeutic modalities and healthcare settings, forming the foundation upon which clinical judgment and decision-making are exercised. However, the main message is that ethical and legal frameworks provide essential guidance but are not sufficient on their own to resolve the complexity of clinical decision-making in AN.

## 4. Ethical Dilemmas in the Treatment of Anorexia Nervosa

### 4.1. The Challenging Nature of Ethical Dilemmas in Anorexia Nervosa

In AN, ethical dilemmas are closely linked to the meaning of eating and food refusal, which are not merely behavioral symptoms but are often intertwined with issues of control, identity, and autonomy. Although ethical codes and legal standards provide clinicians with a sense of stability, consistency, and professional security [48], they are not always sufficient to guide decision-making in the treatment of AN [7]. Ethical dilemmas arise when clinicians are confronted with conflicting principles that cannot be simultaneously fulfilled [66], a situation that occurs routinely in clinical practice with patients diagnosed with AN [14,56,67].

Ethical challenges in the treatment of AN are characterized by fluctuating levels of medical risk and by uncertainty regarding patients’ values, intentions, ambivalence, commitment or resistance to therapy, and decision-making capacity. Consequently, these dilemmas tend to recur and evolve over time in everyday clinical practice, requiring ongoing reassessment rather than definitive resolution [68]. Importantly, such dilemmas do not arise solely at the level of the individual clinician but unfold within a multilayered interpersonal and institutional context. Ethical tensions are embedded within therapeutic teams, shaped by different professional roles and thresholds of acceptable risk, and frequently involve patients’ families or significant others. Institutional policies and healthcare organizations further structure the ethical landscape within which clinical judgment is exercised. Ethical decision-making thus can be considered as a collective and contextual process, shaped through continuous communication, negotiation, and shared responsibility across multiple domains [68].

Legal frameworks, moreover, tend to be reactive rather than anticipatory, offering limited support for the numerous “grey-zone” decisions that precede or follow formal legal intervention [49,52]. Finally, neither ethical codes nor legal frameworks adequately address the internal cognitive and emotional processes through which clinicians interpret and apply these rules in practice [16]. As a result, strict reliance on rule-based approaches risks fostering an illusion of certainty in a clinical context fundamentally characterized by uncertainty [17,69].

### 4.2. Core Ethical Dilemmas in Clinical Practice

Several ethical dilemmas in the treatment of AN are identified in the literature. Among the most prominent are the conflicts between autonomy and protection of life and health, therapeutic alliance and coercive intervention, informed consent and impaired decision-making capacity, and confidentiality and safety. Each of these dilemmas encompasses multiple interrelated and context-sensitive considerations. These dilemmas may also be understood within a broader bioethical framework, reflecting tensions between core ethical principles.

### 4.3. Autonomy vs. Protection

Clinicians must determine when respect for a patient’s autonomy remains ethically defensible and when protection becomes a clinical obligation. This dilemma is complicated by fluctuating decision-making capacity and by patients’ ability to formulate seemingly coherent arguments for refusing treatment despite severe medical risk. In AN, respect for autonomy is further challenged by the ego-syntonic nature of the disorder, as expressed wishes may be closely aligned with illness-driven beliefs rather than enduring and stable over time personal values. Respect for autonomy may also involve consideration of patients’ previously expressed values and preferences over time, particularly in conditions where decision-making capacity may fluctuate [7,47,70].

### 4.4. Therapeutic Alliance vs. Coercive Intervention

Involuntary hospitalization, nasogastric feeding, or other legal measures may be lifesaving in the short term and, in some cases, unavoidable. However, such interventions may undermine the therapeutic alliance that is central to long-term recovery. This dilemma reflects the tension between interventions aimed at immediate survival and their potential impact on trust, motivation, and continuity of care, thereby increasing the risk of disengagement or dropout [14,45,60,61,62].

### 4.5. Informed Consent vs. Impaired Capacity

Although informed consent is a cornerstone of ethical practice, its validity may be compromised in AN by distorted beliefs, denial of illness severity, and cognitive inflexibility. Clinicians must therefore continuously assess whether treatment refusal reflects authentic values or illness-driven distortions, even when information has been adequately provided [14,15,27].

### 4.6. Confidentiality vs. Safety

Decisions regarding information sharing with families or other professionals involved often give rise to a dilemma between confidentiality and patient safety. Breaching confidentiality may be legally permissible and clinically necessary, yet ethically distressing and potentially damaging to trust. In AN, this dilemma is further complicated by the central role of control as a core characteristic of the disorder, as well as by family dysfunctions which, in some cases, may contribute to the onset and maintenance of the illness [13,28].

## 5. Therapist Cognitive and Emotional Processes

Therapists inevitably bring their own patterns of thinking, emotional responses, and personal moral values into the decision-making process [16,17]. From a cognitive perspective, every event leads to automatic thoughts, which in turn lead to emotional reactions and finally influence behavior. In this context, emotional responses are conceptualized within a cognitive framework as closely linked to, and often arising from, automatic thoughts and underlying schemas. This perspective does not encompass the full range of psychological theories of emotion but provides a specific lens for examining clinicians’ internal processes in decision-making.

Automatic thoughts are typically rapid, emotionally charged, and experienced as self-evident, often arising outside deliberate awareness. Within cognitive theory, automatic thoughts are understood as surface-level manifestations of more enduring cognitive structures, commonly described as core beliefs and schemas [71,72]. Schemas are considered as stable cognitive structures consisting of core beliefs and assumptions that organize how individuals perceive, interpret, and respond to internal and external experiences [73,74]. At a deeper cognitive level, schemas can be activated and in turn give rise to corresponding automatic thoughts [75,76]. This process is frequently mediated by cognitive distortions, which are systematic biases in information processing that lead clinicians to interpret clinical situations in a selective or exaggerated manner, often in ways that confirm the activated schema [16,77,78]. The activation of these cognitive processes is closely associated with emotional responses, including anxiety, fear, frustration, helplessness, and anger, or even moral distress [79].

These cognitive and emotional processes may also be understood in relation to the literature on heuristics and cognitive biases in clinical decision-making [80,81]. For example, clinicians may be influenced by confirmation bias, selectively attending to information that supports concerns about risk or responsibility, or by omission–commission bias, where the perceived burden of acting (e.g., involuntary hospitalization) may outweigh the risks associated with inaction. Such processes are particularly relevant in high-risk and uncertain clinical contexts, where rapid judgments are required [81].

When considered in combination, these cognitive and emotional processes indicate that ethical decision-making is not a purely rational or rule-based activity. Rather, it develops as a dynamic and internally mediated process, in which clinicians continuously interpret ethical principles through their own cognitive schemas, emotional states, and moral values. Recognizing and reflecting upon these internal processes constitutes a necessary foundation for ethically informed and clinically responsible decision-making [18]. The reflective framework synthesized in the following section examines how these internal processes interact with ethical and legal constraints and shape clinical judgment.

## 6. A Reflective Framework for Ethical Decision Making in Anorexia Nervosa

The framework presented in this section synthesizes the above-mentioned levels of the decision-making process and outlines a temporal sequence through which these processes typically unfold, conceptualizing ethical decision-making in AN as a process that is sequential yet non-linear. For clarity, these processes can be described across several analytically distinct but interrelated levels.

Ethical decision-making is initiated by clinical events that require a response from the clinician and may activate an ethical dilemma. Such dilemmas are conceptualized as internal conflicts between competing ethical principles. At an initial internal level, clinicians may experience automatic thoughts, schema activation, cognitive distortions and biased interpretations, leading to emotional responses, and varying degrees of moral tension. These processes are activated rapidly and often outside immediate awareness, shaping how clinical risk, responsibility, and available options are initially perceived.

At a subsequent level, these cognitive and emotional responses function as internal filtering processes through which ethical and legal considerations are first interpreted. They determine which aspects of the dilemma become salient, how competing ethical principles are weighted, and how uncertainty is experienced. Importantly, this internal processing typically precedes deliberate consultation of ethical codes or legal standards and may constrain or expand the range of perceived clinical options.

At another level of the process, ethical and legal principles are brought into awareness and function as external constraints that shape the boundaries within which clinical decision-making occurs. Ethical concepts such as autonomy, beneficence, nonmaleficence, and justice, together with legal standards concerning capacity, consent, compulsory treatment, confidentiality, and duty of care, inform clinicians’ deliberations by defining what must be considered and which actions are permitted or required.

Across these levels, supervision and reflective support constitute integral components of the decision-making process. Supervision provides a structured context in which clinicians can examine ethical dilemmas beyond the immediacy of clinical pressure, reflect on their own cognitive and emotional responses with peers, and revisit decisions over time.

Clinical judgment ultimately emerges through a process of reflective integration. This process involves the deliberate consideration of the ethical dilemma arising in everyday clinical circumstances, the relevant ethical and legal constraints, and the clinician’s internal cognitive and emotional responses. Through reflection, previously implicit assumptions, emotional reactions, and schema-driven interpretations may be brought into awareness, allowing for more differentiated consideration of competing ethical demands.

Recurrent clinical events may give rise to new ethical dilemmas, or the same dilemma may reappear over time. Clinical decisions emerging from this process are understood as context-sensitive and provisional rather than definitive. As the therapeutic process evolves, earlier judgments may require reconsideration or modification.

An illustrative application of the proposed framework to a common clinical scenario in AN is provided in Table 1.

## 7. Discussion

This article examines how clinicians’ internal cognitive and emotional processes shape ethical decision-making in the treatment of AN, by integrating established knowledge on ethical principles and legal standards with insights from cognitive theory and reflective practice.

The proposed framework is derived from the comprehensive synthesis of temporally sequential levels and rests on the assumption that clinicians’ internal processes frequently operate implicitly and may substantially influence clinical judgment. Automatic thoughts initially emerged in response to ethical dilemmas and reinforced by previous experiences may shape clinicians’ immediate emotional responses and initial clinical reactions before deliberate ethical reflection or consultation of ethical and legal standards occurs [18]. In high-risk situations, such thoughts may include fears of personal responsibility, such as “the patient will deteriorate, and it will be my fault”; concerns about professional consequences, such as “I may lose my job” or “I will be perceived as incompetent”; or apprehensions regarding relational outcomes, such as “I will upset the patient” or “the patient will disengage from treatment”.

Schema-driven processing further contributes to this dynamic by shaping how ethical situations are interpreted at a deeper cognitive level. Therapists who hold rigid or absolute core beliefs may experience heightened internal conflict when these schemas are activated in clinical practice [82]. Such schemas may include beliefs centered on excessive responsibility, such as “I am personally responsible for preventing harm at all costs”; professional adequacy, such as “If a patient deteriorates or disengages, it means I have failed as a clinician”; or relational obligation, such as “Causing distress or rupture in the therapeutic relationship is unacceptable”.

Cognitive biases may shape ethical reasoning by influencing how information is selectively attended to, interpreted, and remembered [75,80]. Overestimation of responsibility may generate automatic thoughts such as “If the patient deteriorates, it will be my fault,” reinforcing schemas of excessive personal responsibility. Catastrophizing may amplify perceived threat and urgency through thoughts like “Any delay will have irreversible consequences,” while dichotomous thinking may frame ethical choices as absolute and mutually exclusive: “Either I respect autonomy and allow harm, or I intervene and violate ethics”. Confirmation bias may further lead clinicians to privilege information that supports pre-existing beliefs, such as interpreting treatment refusal solely as evidence of impaired capacity, while disregarding information that challenges these assumptions.

Through these mechanisms, cognitive biases contribute to a self-reinforcing cycle in which automatic thoughts confirm underlying schemas, further constraining ethical deliberation, and promoting familiar or defensive patterns of decision-making rather than flexible, context-sensitive ethical reasoning [76] which could potentially influence diagnosis and treatment decisions [81]. In ethically charged contexts, as in the treatment of AN, these cognitive procedures may intensify ethical tension prior to reflective deliberation and result in biased attention toward certain risks or ethical priorities, while marginalizing others, thereby narrowing the range of perceived ethical options and predispose clinicians toward prioritizing particular ethical principles over others before conscious ethical reflection occurs.

Emotional responses associated with these cognitive processes may be intense, given the high-risk nature of clinical decisions in AN. Heightened emotional activation can interfere with reflective capacity and ethical clarity, particularly when uncertainty is experienced as intolerable [83]. Compassion fatigue is an emotional aspect experienced by healthcare professionals faced with intense clinical situations [84]. Moral distress also represents a salient manifestation of this emotional burden, emerging when clinicians perceive an ethically appropriate course of action but feel constrained by legal, institutional, or clinical factors [79,85,86]. In the context of AN, moral distress may emerge when clinicians are required to enforce compulsory treatment, respect treatment refusal despite medical risk, or navigate conflicting expectations from families, teams, and legal systems [87]. Over time, unresolved moral distress may contribute to emotional exhaustion, defensive practice, and diminished ethical sensitivity.

Within this approach, supervision facilitates the externalization and examination of cognitive assumptions, emotional responses, and moral tensions that may influence ethical judgment beyond immediate clinical pressure. Supervision may function as a reflective space in which fast, automatic modes of thinking are slowed, enabling the activation of more deliberate and analytical processing. In this way, it supports a shift from intuitive, experience-based judgments toward more reflective and context-sensitive clinical reasoning. Supervisory teams may support emotional containment, reduce isolation, and help prevent the consolidation of rigid or defensive decision-making patterns. It also supports shared responsibility and interdisciplinary dialogue, which are particularly relevant in high-risk clinical contexts. Reflective support thus contributes to the ongoing reassessment of clinical judgment as circumstances change [88]. The proposed framework may also be applied prospectively, allowing clinicians to identify personal cognitive and emotional triggers—such as responses to clinical deterioration or lack of improvement—and to develop reflective strategies in advance. In this way, supervision may support anticipatory awareness and enhance preparedness for ethically challenging situations.

The importance of reflective capacity in clinical decision-making has been widely recognized. Reflective capacity may function as a regulatory process through which automatic thoughts and associated emotional responses are identified, examined, and potentially re-evaluated. This process allows clinicians to move from rapid, intuitive responses toward more deliberate and context-sensitive ethical judgment. Beyond the field of AN, a substantial body of literature indicates that clinical judgment emerges from the interaction between fast, automatic cognitive processes and slower, deliberative reasoning, with heuristic thinking which may give rise to cognitive biases exerting a strong influence, particularly under conditions of time pressure, emotional activation, perceived responsibility, uncertainty, and high clinical risk [77,78,89,90]. These automatic processes are activated rapidly and largely outside conscious awareness, shaping judgment before explicit deliberation occurs [76,80,91]. Foundational work on professional judgment has emphasized that clinical decision-making cannot be reduced to the mechanical application of rules but unfolds in contexts characterized by uncertainty and complexity, requiring ongoing reflection during and after action [17]. Within this perspective, reflective and mindful approaches have been proposed as ways of addressing ineffective or harmful clinical practices through increased awareness of clinicians’ thoughts, emotions, and values [92,93]. In psychotherapy, empirical work further demonstrates that therapists’ cognitive biases influence case formulation, risk assessment, and intervention choices, often outside awareness [16], while greater reflective capacity and self-awareness have been associated with improved professional judgment, ethical sensitivity, and responsiveness to complex clinical situations [94,95].

Several models and frameworks have been proposed to support ethical decision-making in the treatment of eating disorders, particularly in complex or severe presentations. These approaches emphasize structured ethical deliberation, collaborative decision-making, and the use of reflective or procedural tools to support clinicians navigating high-risk and morally challenging situations, including treatment refusal and prolonged illness trajectories [14,45,47,96]. While such frameworks provide valuable guidance for team-based deliberation and institutional decision-making, they primarily address how ethical decisions are discussed, negotiated, or justified within clinical and organizational contexts [14,45,47]. In contrast, the present framework focuses on the moment-to-moment cognitive and emotional processes through which clinicians interpret and apply these principles in practice. By explicitly mapping elements such as automatic thoughts, underlying schemas, and emotional responses, the model seeks to account for how ethical judgment is shaped in real time, rather than assuming a fully rational or procedural decision-making process. This shift from a primarily normative and procedural perspective to a process-oriented and psychologically informed account constitutes the main contribution of the present work. This focus differs from existing procedural or team-based models, which primarily address how decisions should be structured or justified, rather than how they are cognitively and emotionally mediated in practice. In addition, the use of a familiar and accessible cognitive framework may facilitate clinicians’ identification with the described processes, thereby enhancing its practical applicability in everyday clinical settings.

The present framework is grounded in a cognitive perspective, focusing on clinicians’ internal cognitive and emotional processes as mediators of ethical decision-making. The choice to adopt a cognitive framework in the present article reflects the intention to provide a structured and widely applicable conceptual language for examining clinicians’ internal processes across different therapeutic settings. Its strength lies in offering a structured and operationalized account of these processes, which may be more readily accessible in everyday clinical practice. In particular, the framework reflects common patterns of clinical experience, allowing clinicians to recognize their own cognitive and emotional responses within ethically challenging situations, thereby supporting more engaged and reflective decision-making.

However, alternative theoretical approaches conceptualize these processes differently and employ distinct conceptual languages, often emphasizing relational, affective, and unconscious dimensions of clinicians’ responses rather than structured cognitive constructs. Psychodynamic perspectives, for example, conceptualize clinicians’ internal responses as shaped by unconscious relational processes, including transference and countertransference, as well as resistance and the influence of past relational experiences, which may influence how clinicians perceive risk, responsibility, and patient engagement in ethically complex situations, often outside of immediate conscious awareness [97]. Relational and psychosocial approaches, in turn, highlight the role of empathy, interpersonal dynamics, and broader contextual and developmental factors, including patients’ life histories and social environments. Each theoretical orientation therefore offers its own framework for understanding clinical decision-making, and these perspectives may be considered complementary rather than mutually exclusive in capturing the complexity of ethical dilemmas in AN. Therefore, the framework should be understood as one possible lens among others, rather than a comprehensive account of ethical decision-making in AN. In clinical practice, these processes are embedded within ongoing interactions with patients, where issues such as resistance, ambivalence, and relational dynamics may further influence decision-making.

The proposed framework has implications for clinical practice, education and supervision, particularly in contexts characterized by ethical uncertainty and high clinical risk. At the level of clinical practice and supervision, the framework highlights the importance of creating reflective spaces in which clinicians can systematically examine how their internal experiences shape ethical judgment over time. Within interdisciplinary eating disorder teams, the use of a shared conceptual language for discussing cognitive, emotional, and ethical processes may facilitate dialogue, enhance mutual understanding across professional roles, and promote collective responsibility in ethically complex situations. At the level of education and training, the present synthesis underscores the importance of training programs in the care of AN and psychotherapy that incorporate reflective exercises, case-based discussions, and reflective teaching practices explicitly addressing how cognitive biases, emotional activation, and moral intuitions influence ethical reasoning in clinical practice. Such approaches may foster ethical sensitivity and reflective capacity early in professional development.

## 8. Limitations

Several limitations should be acknowledged when interpreting the present synthesis. This article adopts a conceptual and narrative approach and does not present empirical data. Empirical validation is further required in clinical settings to assess its applicability and impact on decision-making. Accordingly, the proposed framework is intended to support reflection and conceptual understanding of ethical decision-making, rather than to offer procedural guidance, decision-making algorithms, or prescriptive recommendations. The synthesis focuses primarily on clinicians’ internal cognitive and emotional processes and does not directly incorporate patients’ perspectives or lived experiences. It is also grounded in cognitive theory and does not explicitly engage with alternative theoretical perspectives that conceptualize therapists’ internal processes through different constructs, such as countertransference. Further empirical research is therefore needed to examine how clinicians’ cognitive and emotional processes influence ethical judgment in practice and to explore how reflective interventions may affect ethical decision-making over time. Consequently, its grounding in cognitive theory may oversimplify the complexity of clinical experience and underrepresent relational, unconscious, and contextual dimensions that are emphasized in other theoretical approaches.

In addition, the framework is informed by the perspectives of clinicians, which may introduce a degree of professional bias; the inclusion of patient perspectives could further enrich its development. Practical implementation of reflective processes may also be constrained in healthcare systems with limited time and resources. Finally, the framework does not explicitly address cultural variability, particularly in the interpretation of autonomy, which may influence ethical decision-making across different contexts.

## 9. Conclusions

In conclusion, the present synthesis does not aim to propose decision-making tools or prescriptive guidance, but to make explicit the internal cognitive schemas, emotional responses, and moral intuitions through which ethical and legal standards are interpreted and applied in clinical practice. Ethical decision-making is conceptualized not as a linear progression from ethical principles to clinical action, but as a dynamic and iterative process unfolding at the intersection of normative constraints, clinical risk, and clinicians’ internal experience. By foregrounding these internal processes, the framework highlights reflective awareness as a foundational prerequisite for ethically responsible clinical judgment in the treatment of AΝ. Finally, the present framework should not be used to justify clinical actions based on subjective cognitive or emotional processes. Rather, it is intended to enhance reflective awareness, while final decisions must remain anchored in objective clinical, ethical, and legal criteria.

## Figures and Tables

**Table 1:** Illustrative application of the reflective framework for ethical decision-making in anorexia nervosa.

Framework Levels	Illustrative Clinical Example
Clinical event	A patient with anorexia nervosa presents with significant weight loss and medical instability while refusing further nutritional intervention.
Emerged dilemma	The clinician experiences a conflict between respecting the patient’s expressed refusal of treatment and the perceived obligation to prevent serious physical harm.
Automatic thoughts	“The patient will be deteriorate”, “it will be my fault.”
Core beliefs/schema	A schema of excessive responsibility is activated “I must prevent harm at all costs, even if this overrides the patient’s wishes”.
Distortions	Catastrophizing “Any delay will have irreversible consequences” and dichotomous thinking “Either I intervene forcibly, or I am acting unethically”.
Emotions	Elevated anxiety, fear of negative outcomes, guilt related to perceived responsibility, and moral distress in the face of ethical uncertainty are experienced.
Awareness of ethical codes	Ethical principles such as beneficence and nonmaleficence are foregrounded, while respect for autonomy is perceived as secondary in the context of medical risk.
Awareness of legal principles	Consideration of legal standards regarding decision-making capacity and the conditions under which compulsory treatment may be justified.
Supervision	In supervision, the clinician explores personal fears of blame and responsibility and examines how these reactions shape the framing of the dilemma.
Reflection	Reflective deliberation allows reconsideration of alternative clinical options, including graded interventions and continued engagement alongside medical monitoring.
Context based decision	A context-sensitive decision is made that balances medical safety with efforts to preserve therapeutic alliance and acknowledging uncertainty.
Recurrent clinical event	As the patient’s condition and engagement fluctuate over time, similar dilemmas re-emerge, requiring renewed reflection and adjustment of clinical judgment.

Note: The illustrative example presented in this table does not constitute prescriptive guidance or a ‘best’ solution to ethical dilemmas. It is intended solely to demonstrate one possible trajectory of ethical reflection, among multiple ethically defensible pathways, depending on clinical context.

## Data Availability

No new data were created or analyzed in this study. Data sharing is not applicable to this article.
